# Supplementary material for: Propyl Gallate Attenuates Methylglyoxal-Induced Alzheimer-like Cognitive Deficits and Neuroinflammation in Mice
Source: Int J Mol Sci. 2026 Jan 4;27(1):511. doi: 10.3390/ijms27010511 (PMC12786693; doi:10.3390/ijms27010511)
Supplement: Supplementary file 1 [file ijms-27-00511-s001.zip › ijms-4068951-supplementary.pdf]

Supplementary Materials

Propyl Gallate Attenuates Methylglyoxal-Induced Alzheimer-like Cognitive Deficits and Neuroinflammation in Mice

Hui-Yun Tsai [1,2], Jing Qiu [3], Han-Wei Liao [3], Chi-I Chang [4], Yu-Hsiang Chen [5], Chi-Tang Ho [6], Yu-Kuo Chen [3,*]

[1] Department of Nutrition and Health Science, Fooyin University, Kaohsiung 831301, Taiwan; rhytsai@gmail.com

[2] Aging and Disease Prevention Research Center, Fooyin University, Kaohsiung 831301, Taiwan

[3] Department of Food Science, National Pingtung University of Science and Technology, Pingtung 912301, Taiwan; asus0979694098@gmail.com (J.Q.); b123599272@gmail.com (H.-W.L.)

[4] Department of Biological Science and Technology, National Pingtung University of Science and Tech-nology, Pingtung 912301, Taiwan; changchii@mail.npust.edu.tw

[5] Department of Medical & Molecular Genetics, Indiana University School of Medicine, Indianapolis, IN 46202, USA; yuhschen@iu.edu

[6] Department of Food Science, Rutgers, The State University of New Jersey, New Brunswick, NJ 08901, USA; ctho@sebs.rutgers.edu

[*] Correspondence: chenyk@mail.npust.edu.tw

Table S1

The change of body weight in C57BL/6J mice during the experiment.

Group	Body weight (g)								
	week 1	week 2	week 3	week 4	week 5	week 6	week 7	week 8	week 9
Control	24.40 ± 0.33	24.43 ± 0.33	24.82 ± 0.43	25.14 ± 0.44	25.36 ± 0.45	25.64 ± 0.48	26.67 ± 0.53	26.20 ± 0.52	26.64 ± 0.50
MG	24.23 ± 0.60	24.73 ± 0.60	25.16 ± 0.59	25.53 ± 0.60	25.58 ± 0.58	25.89 ± 0.58	26.19 ± 0.59	26.45 ± 0.62	26.87 ± 0.66
LPG	24.35 ± 0.49	24.64 ± 0.48	25.02 ± 0.52	25.19 ± 0.58	25.12 ± 0.52	25.60 ± 0.52	26.23 ± 0.61	25.86 ± 0.60	26.08 ± 0.62
MPG	24.45 ± 0.39	24.57 ± 0.29	24.85 ± 0.28	25.21 ± 0.30	25.00 ± 0.33	25.44 ± 0.26	25.92 ± 0.35	25.74 ± 0.35	26.16 ± 0.36
HPG	24.52 ± 0.34	24.45 ± 0.19	24.44 ± 0.33	24.60 ± 0.28	24.63 ± 0.28	24.99 ± 0.37	24.90 ± 0.33	25.06 ± 0.47	25.56 ± 0.37

Mice were treated with PG and 1% MG in H_2O for 9 weeks and each value represents mean ± SEM (n=10 per group). Control: vehicle; MG: 1% MG in H_2O; LPG: 1% MG in H_2O + 20 mg/kg/day PG; MPG: 1% MG in H_2O + 40 mg/kg/day PG; HPG: 1% MG in H_2O + 100 mg/kg/day PG.

Table S2

The food and water intake in C57BL/6J mice during the experiment.

	Control	MG	LPG	MPG	HPG
Food intake (g/mice/day)	3.29 ± 0.13	3.18 ± 0.12	3.17 ± 0.11	3.10 ± 0.12	2.95 ± 0.20
Water intake (mL/mice/day)	5.61 ± 0.25[a]	4.73 ± 0.33[b]	4.67 ± 0.27[b]	4.75 ± 0.33[b]	4.67 ± 0.40[b]

Mice were treated with PG and 1% MG in H_2O for 9 weeks and each value represents mean ± SEM (n=10 per group). The letters (a,b) indicate statistically significantly at p<0.05. Control: vehicle; MG: 1% MG in H_2O + vehicle; LPG: 1% MG in H_2O + 20 mg/kg/day PG; MPG: 1% MG in H_2O + 40 mg/kg/day PG; HPG: 1% MG in H_2O + 100 mg/kg/day PG.

Table S3

The changes of organ weight in C57BL/6J mice.

	Control	MG	LPG	MPG	HPG
Brain (g)	0.40 ± 0.00	0.41 ± 0.01	0.40 ± 0.00	0.39 ± 0.01	0.38 ± 0.01
Liver (g)	1.05 ± 0.03	1.02 ± 0.02	1.03 ± 0.02	1.06 ± 0.02	1.02 ± 0.01
Intestine (g)	1.96 ± 0.06	2.02 ± 0.06	1.96 ± 0.06	1.91 ± 0.04	1.82 ± 0.06
Kidney (g)	0.29 ± 0.01	0.31 ± 0.01	0.29 ± 0.01	0.28 ± 0.01	0.28 ± 0.01
Spleen (g)	0.05 ± 0.00	0.06 ± 0.00	0.06 ± 0.00	0.05 ± 0.00	0.05 ± 0.00
Relative Brain (%)	1.73 ± 0.03	1.72 ± 0.04	1.74 ± 0.03	1.68 ± 0.04	1.73 ± 0.04
Relative Liver (%)	4.48 ± 0.09	4.36 ± 0.11	4.44 ± 0.08	4.56 ± 0.04	4.56 ± 0.09
Relative Intestine (%)	8.34 ± 0.27	8.59 ± 0.20	8.46 ± 0.22	8.22 ± 0.21	8.09 ± 0.19
Relative Kideny (%)	1.21 ± 0.05	1.30 ± 0.03	1.24 ± 0.02	1.19 ± 0.01	1.24 ± 0.03
Relative Spleen (%)	0.22 ± 0.01	0.23 ± 0.01	0.25 ± 0.01	0.22 ± 0.01	0.21 ± 0.01

Mice were treated with PG and 1% MG in H2O for 9 weeks and each value represents mean ± SEM (n=10 per group). Value are absolute wet weight of organ (g) and relative organ weight (%, per body weight). Control: vehicle; MG: 1% MG in H_2O + vehicle; LPG: 1% MG in H_2O + 20 mg/kg/day PG; MPG: 1% MG in H_2O + 40 mg/kg/day PG; HPG: 1% MG in H_2O + 100 mg/kg/day PG.

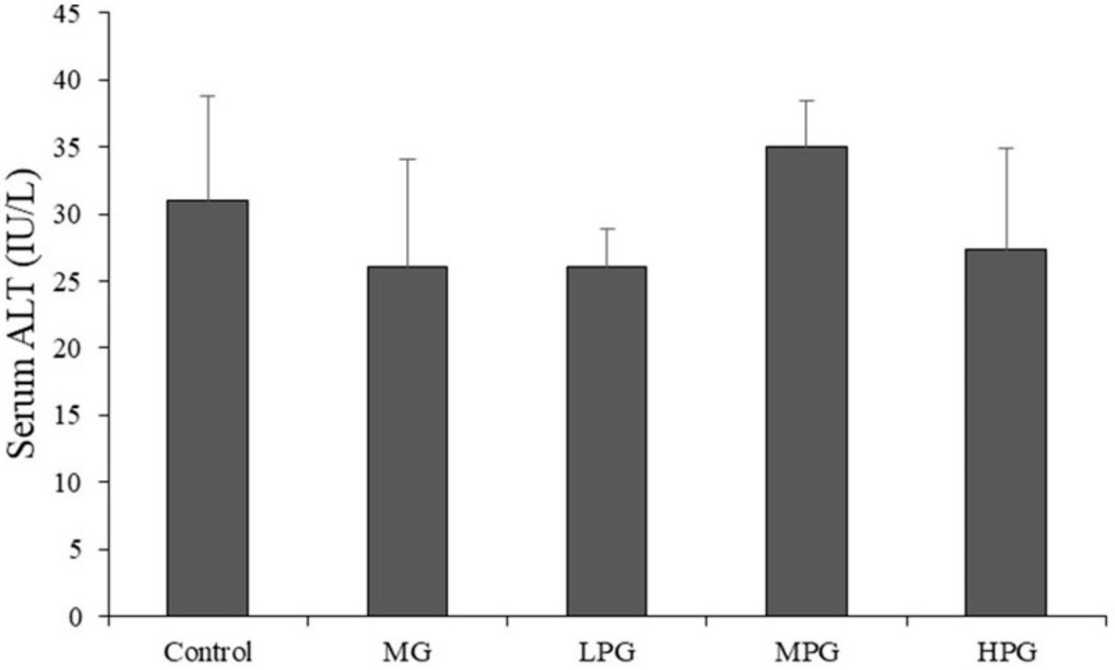

Figure S1. Effects of PG on serum ALT in C57BL/6J mice. Mice were treated with PG and 1% MG in H_2O for 9 weeks. The level of serum ALT was measured at 8 weeks in mice. Results are expressed as mean ± SEM (n=10 per group). Control: vehicle; MG: 1% MG in H_2O + vehicle; LPG: 1% MG in H_2O + 20 mg/kg/day PG; MPG: 1% MG in H_2O + 40 mg/kg/day PG; HPG: 1% MG in H_2O + 100 mg/kg/day PG.
